# Supplementary material for: Prognostic Value of C-Reactive Protein and Albumin in Neurocritically Ill Patients with Acute Stroke
Source: J Clin Med. 2022 Aug 29;11(17):5067. doi: 10.3390/jcm11175067 (PMC9457411; doi:10.3390/jcm11175067)
Supplement: Supplementary file 1 [file jcm-11-05067-s001.zip › jcm-1858848-supplementary.pdf]

Supplementary Materials

Table S1. Comparison of serum CRP and Albumin levels between stroke patients with malignancy and those without malignancy.

	**Malignancy**	**Non-malignancy**	***p***
CRP, mg/L	5.2 ± 7.4	4.0 ± 6.4	0.177
Albumin, g/L	3.4 ± 0.5	3.6 ± 0.5	0.002
CAR	175.6 ± 268.6	130.8 ± 241.8	0.160
ΔCRP	2.7 ± 6.7	1.9 ± 4.6	0.349
CRPc	-15.9 ± 310.5	-8.1 ± 224.9	0.824
D-dimer, μg/mL	14.4 ± 17.7	6.6 ± 6.9	0.068
Fibrinogen, mg/dL	298.4 ± 140.0	308.9 ± 121.1	0.733
Lactate dehydrogenase, U/L	500.0 ± 309.6	466.1 ± 209.5	0.506
ICU mortality	4 (4.2%)	12 (5.7%)	0.780
In-hospital mortality	13(13.5%)	27(12.8%)	0.999

Data are presented as means ± standard deviations.

CRP, C-reactive protein; CAR, ratio of CRP to Albumin; ΔCRP, delta CRP; CRPc, clearance of CRPs.

Table S2. Comparison of serum CRP and Albumin levels according to stroke subtypes.

	Cerebral infarction	Intracerebral hemorrhage	Subarachnoid hemorrhage	***p***
CRP, mg/L	6.4 ± 8.4	5.4 ± 7.6	2.9 ± 4.8	0.005
Albumin, g/L	3.2 ± 0.6	3.5 ± 0.6	3.6 ± 0.5	0.156
CAR	249.4 ± 415.4	180.7 ± 285.2	90.4 ± 165.8	0.005
ΔCRP	1.2 ± 7.6	2.6 ± 6.3	1.7 ± 3.5	0.284
CRPc	24.9 ± 96.7	-30.2 ± 315.5	13.1 ± 147.0	0.320
ICU mortality	1 (11.1)	21 (12.4)	12 (9.4)	0.720
In-hospital mortality	1 (11.1)	25 (14.7)	14 (10.9)	0.623

Data are presented as means ± standard deviations.

CRP, C-reactive protein; CAR, ratio of CRP to Albumin; ΔCRP, delta CRP; CRPc, clearance of CRPs.

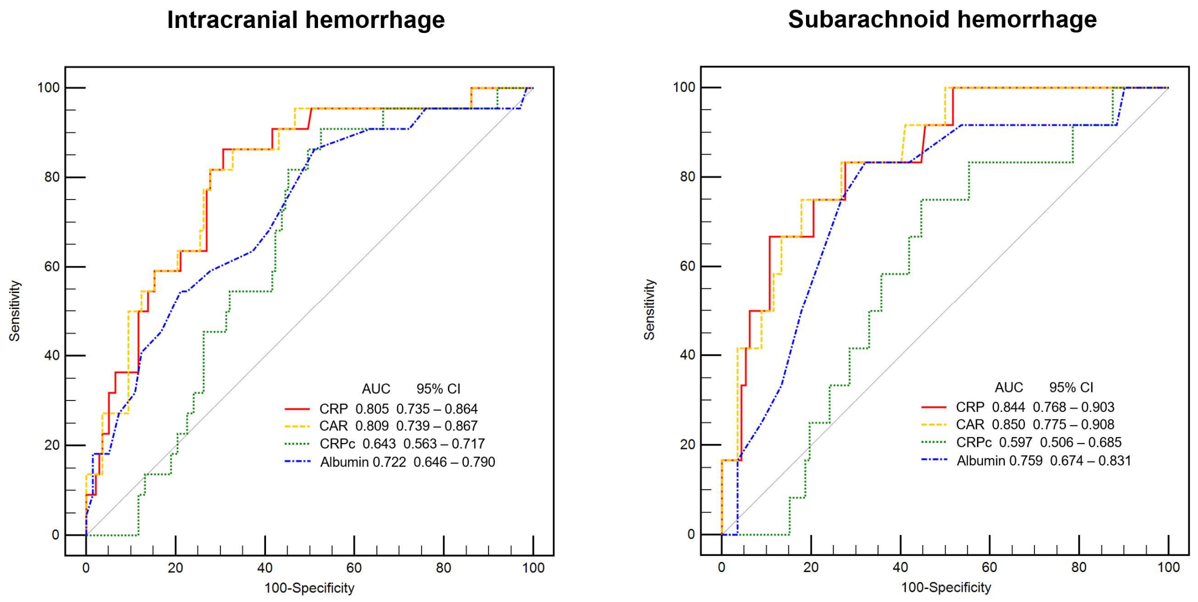

Figure S1. Receiver operating characteristic curves for predicting in-hospital mortality using levels of C-reactive protein (CRP), CRP to albumin ratio (CAR), CRP clearance (CRPc) and albumin according to intracranial hemorrhage and subarachnoid hemorrhage. AUC, area under the curve; CI, confidence interval.
